# CBL0137 increases the targeting efficacy of Rovalpituzumab tesirine against tumour-initiating cells in small cell lung cancer

**DOI:** 10.1038/s41416-020-01192-x

**Published:** 2020-12-01

**Authors:** Daniel J. Lindner, Gary Wildey, Yvonne Parker, Afshin Dowlati, George R. Stark, Sarmishtha De

**Affiliations:** 1 Department of Translational Hematology and Oncology Research, Cleveland Clinic, Cleveland, OH USA; 2 University Hospitals Seidman Cancer Center, Case Comprehensive Cancer Center, Case Western Reserve University, Cleveland, OH USA; 3 Department of Cancer Biology, Cleveland Clinic Lerner Research Institute, Cleveland, OH USA

**Keywords:** Small-cell lung cancer, Targeted therapies

## Abstract

Small cell lung cancer (SCLC) is characterised by high relapse rates. Tumour-initiating cells (TICs) are responsible for drug resistance and recurrence of cancer. Rovalpituzumab tesirine (Rova-T), a potent humanised antibody–drug conjugate, selectively targets delta-like protein 3, which is highly expressed in SCLC TICs. The experimental drug CBL0137 (CBL) inhibits the histone chaperone FACT (facilitates chromatin transcription), which is required for the expression of transcription factors that are essential for TIC maintenance. Rova-T and CBL each target SCLC TICs as single agents. However, acquired or intrinsic resistance to single agents is a major problem in cancer. Therefore, we investigated the potential effect of combining Rova-T and CBL in SCLC to eradicate TICs more effectively. Our preclinical studies report a novel and highly translatable therapeutic strategy of dual targeting TICs using Rova-T in combination with CBL to potentially increase survival of SCLC patients.

## Background

Small cell lung cancer (SCLC) has very high mortality because of its high relapse rate after standard-of-care therapies, coupled with a lack of effective second-line therapies. Tumour-initiating cells (TICs) within most solid tumours, including SCLC,^1^ are important contributors to disease recurrence, metastasis and therapeutic resistance.^2,3^ TICs can be identified by a high expression of specific marker proteins, such as CD133, compared with the bulk tumour cell population.^4^

Rovalpituzumab tesirine (Rova-T) is an antibody–drug conjugate (ADC) that comprises a humanised anti-delta-like protein 3 (DLL3) monoclonal antibody attached to a DNA-damaging pyrrolobenzodiazepine toxin.^5^ Rova-T is considered to be the first biomarker-directed treatment for SCLC and is particularly effective against TICs.^5^

CBL0137 (CBL) targets FACT (facilitates chromatin transcription), a histone chaperone that is expressed at high levels in tumours and is required for the expression of transcription factors that are essential for TIC maintenance.^6,7^ Recently, we reported that CBL as a single agent preferentially targets TICs in SCLC and has potent anti-cancer activity against SCLCs when combined with cisplatin.^8^

Thus, the TIC-targeting mechanisms of CBL and Rova-T are entirely different, targeting two different proteins, FACT and DLL3, respectively, that are highly expressed in SCLC TICs. Here, we investigated the therapeutic efficacy of these drugs in combination using both in vitro and patient-derived xenograft (PDX) models of SCLC. PDXs are well recognised as predictors of human cancer biology and patient response to treatment. The objective of these preclinical studies was to report a novel and highly translatable therapeutic strategy of dual targeting TICs that can potentially increase survival of SCLC patients.

## Methods

The CD133^high^ (TIC) and CD133^low^ (non-TIC) cells were generated from NCI-H82 (H82) and NCI-H526 (H526) as described previously.^8^ The PDX tumour (JHU-LX102), derived from a chemotherapy-naive SCLC patient, was a generous gift from Dr. Charles M. Rudin (Memorial Sloan Kettering Cancer Center, New York). NOD.Cg-*Prkdc*^scid^ *Il2rg*^tm1Wjl^/SzJ mice, commonly known as the NOD *scid* IL-2 receptor gamma knockout (NSG) mice, were inoculated in both flanks with 50,000 tumour cells in each and randomised into cohorts of four animals per arm with mean starting tumour volumes of 100 mm^3^. Mice were treated with vehicle control for CBL + IgG control, Rova-T alone, CBL alone or the combination of Rova-T with CBL.^5,8^ Tumour volumes were measured three times a week until vehicle-treated mice reached ~1200 mm^3^, at which time all mice were euthanised using a gradient controlled CO_2_ inhalation, followed by cervical dislocation, and the tumours removed. In a second experiment, tumour-bearing mice were treated with the vehicle controls and the drugs as above, but now each group was treated until tumours reached the maximum size of ~1200 mm^3^. For in vivo limiting dilution assays, cell suspensions from residual tumours harvested from the tumorigenicity studies were inoculated subcutaneously in limiting dilutions (10^3^–10^5^) into naive NSG mice.^9^ The TIC frequency (TIF) was calculated using the ELDA software (http://bioinf.wehi.edu.au/software/elda/).^10^

## Results

### Combination of Rova-T and CBL increases anti-tumour efficacy in vitro and in vivo by decreasing tumour-initiating frequency

**Fig. 1:**
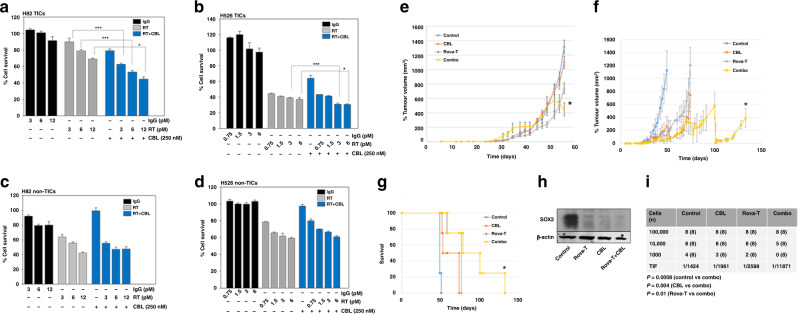
Anti-tumour efficacy of combining Rova-T and CBL in vitro and in vivo. **a** H82 TICs, **b** H526 TICs, **c** H82 non-TICS and **d** H526 non-TICs were seeded at 3000 cells per well in black-walled 96-well plates. The next day cells were treated with IgG control or Rova-T at different concentrations, or CBL, or with Rova-T + CBL. The cell viability after 72 h of treatment was determined using the CyQUANT Fluorescent Assay^8^ and normalised to controls. The experiments were repeated thrice, and each measurement was performed in triplicate. Results are represented by means ± SD. Data were analysed using Student’s *t* test. *P* values of <0.05 are considered statistically significant. *P < 0.05, and ***P < 0.001. **e**, **f** Tumour volumes in mice after treatment with the combination of Rova-T and CBL. Four PDX mice with tumours in both the flanks (*N* = 8) were treated with vehicle controls for CBL + IgG control, Rova-T alone (1.8 mg/kg, i.p.), CBL alone (60 mg/kg, i.v., once per week) or combinations of Rova-T with CBL. Mice were treated with vehicle controls or CBL + IgG control, Rova-T alone, CBL alone or combinations of Rova-T with CBL. **e** Tumour volumes were measured in all groups until they reached ~1200 mm^3^ in the vehicle-treated mice. **f** The treatment at the same doses as above was continued until the tumour volumes reached ~1200 mm^3^ in each group. Tumour volumes (*v*) were calculated using the volume for a prolate spheroid: *v* = 4/3 × *π* × *a*^2^ × b, where *a* is the minor radius and *b* the major radius. Differences between groups were analysed by the Student’s *t* test. The results are represented as means ± SE. * indicates *P* < 0.05 versus the single drug treatment groups. **g** Survival of mice shown in **f** (*P* < 0.05 for the combination vs single drugs alone). **h** SOX2 protein level was determined by Western analysis in the residual tumours derived from mice after treatment with vehicle for CBL + IgG control, or with CBL, Rova-T or Rova-T + CBL. β-Actin was used as a loading control. **i** In vivo limiting dilution assay showing that combining Rova-T and CBL reduced the tumour-initiating frequency (TIF). Mice were scored positive for tumour growth when the tumour size exceeded 200 mm^3^ at 6 months after tumour cell inoculation. The TIF was calculated from *N* = 8 mice per group for each dilution of cells.

Rova-T kills SCLC TICs by targeting DLL3.^5^ We observed a higher expression of DLL3 in TICs of H82 and H526 compared to non-TICs (Supplementary Fig. 1A–C), and treatment with CBL had no effect on DLL3 expression in TICs (Supplementary Fig. 1D), suggesting that CBL does not interfere with Rova-T efficacy by decreasing DLL3 levels. The combination of Rova-T and CBL decreased cell survival in H82 and H526 TICs much better than either drug alone (Fig. 1a, b). Importantly, the drug combination had no additional effect on the sensitivity of non-TICs compared to the single drugs alone (Fig. 1c, d), emphasising the preferential targeting of TICs by these drugs.

We compared the anti-tumour efficacy of Rova-T combined with CBL against single agents in a SCLC PDX model. There was no significant reduction in tumour growth in the mice treated with Rova-T plus CBL until day 50 compared to the groups treated with the individual drugs. However, tumour sizes started decreasing significantly (*P* < 0.05) after day 55 in the combination group (Fig. 1e). Since the vehicle-treated group had already reached the maximum allowed size by that day, we sacrificed the mice in all groups at that time. In a second experiment, tumour-bearing mice were treated with the vehicle controls or drugs at the same doses as above, but in this experiment, the mice in each group were treated until their tumours reached the maximum size of ~1200 mm^3^. Treatment was started on day 31 after inoculation. Rova-T in combination with CBL substantially inhibited tumour growth, compared to Rova-T alone (*P* < 0.05), CBL alone (*P* < 0.05) or vehicle control (*P* < 0.05) (Fig. 1f). We observed that mice treated with combined Rova-T and CBL survived for 133 days, whereas vehicle-treated mice survived for 51 days, and mice treated with either CBL or Rova-T survived for 74–77 days (Fig. 1g). We could also show that the well-established cancer stem cell marker SOX2 level was decreased in the residual tumours from mice treated with CBL and Rova-T, compared to controls, with the drug combination being most effective (Fig. 1h). Rova-T treatment decreased DLL3 expression in residual tumours (Supplementary Fig. 2), indicating that Rova-T kills DLL3-expressing TICs. These results indicate that Rova-T in combination with CBL decreased the growth of SCLC PDX tumours and also increased survival by killing TICs, revealing a novel potent combination therapy for this cancer. We did not observe any toxicity in animals during treatment.

The limiting dilution assay is a rigorous test to quantitate cellular tumour-initiating capacity within a heterogeneous cancer cell population. Both Rova-T^5^ and CBL^7,8^ as single agents reduce tumour-initiating capacity in vivo^4^ or in vitro.^7,8^ To determine whether Rova-T combined with CBL can reduce tumour recurrence by targeting TICs, we performed in vivo limiting dilution assays on residual tumours. Tumours derived from control mice were shown to have a TIF of 1:1424, which was reduced to 1:1961 and 1:2598 in CBL or Rova-T-treated mice, respectively. A substantial reduction in the TIF to 1:11,871 was observed in tumours derived from mice treated with the combination of Rova-T and CBL (Fig. 1i).

## Discussion

SCLCs contain a much higher percentage of TICs than non-SCLCs, 65–75% compared to 15–20%,^11^ and may therefore represent an ideal cancer in which to target TICs, which are relatively insensitive to chemotherapy and seed the growth of newly resistant tumours. While Rova-T and CBL have each been shown to target SCLC TICs as single agents,^5,8^ it is unlikely that any drug will be curative as a single agent. Our findings show that the combination of two different TIC inhibitors, targeting two different proteins, DLL3 and FACT, is much more toxic to SCLC TICs than either drug alone, and this therapeutic strategy is effective in vivo.

Tumour TICs can self-renew, differentiate and give rise to a new tumour. We reveal that the combination of Rova-T and CBL decreases SOX2 and attenuates the in vivo self-renewal capability of SCLC tumours by eradicating TICs, and thereby may also help to counteract relapse.

CBL is currently in the final stages of multicentre phase I clinical trials in advanced or metastatic solid tumours and lymphomas (NCT01905228), and it has not yet exhibited dose-limiting toxicity. Therefore, using CBL in combination with Rova-T may add therapeutic value to disappointing recent results with Rova-T alone in SCLC,^12,13^ and could represent a novel drug combination that can prevent tumour recurrence and yield a more durable response in this cancer.

## Supplementary information

Supplementary material

## Data Availability

All data generated or analysed during this study are included in this article and its supplementary information files.
