# Supplementary material for: CBL0137 increases the targeting efficacy of Rovalpituzumab tesirine against tumour-initiating cells in small cell lung cancer
Source: Br J Cancer. 2020 Dec 1;124(5):893–5. doi: 10.1038/s41416-020-01192-x (PMC7921085; doi:10.1038/s41416-020-01192-x)
Supplement: Supplementary file 1 — Supplementary material [file 41416_2020_1192_MOESM1_ESM.docx]

**Supplementary Figures**


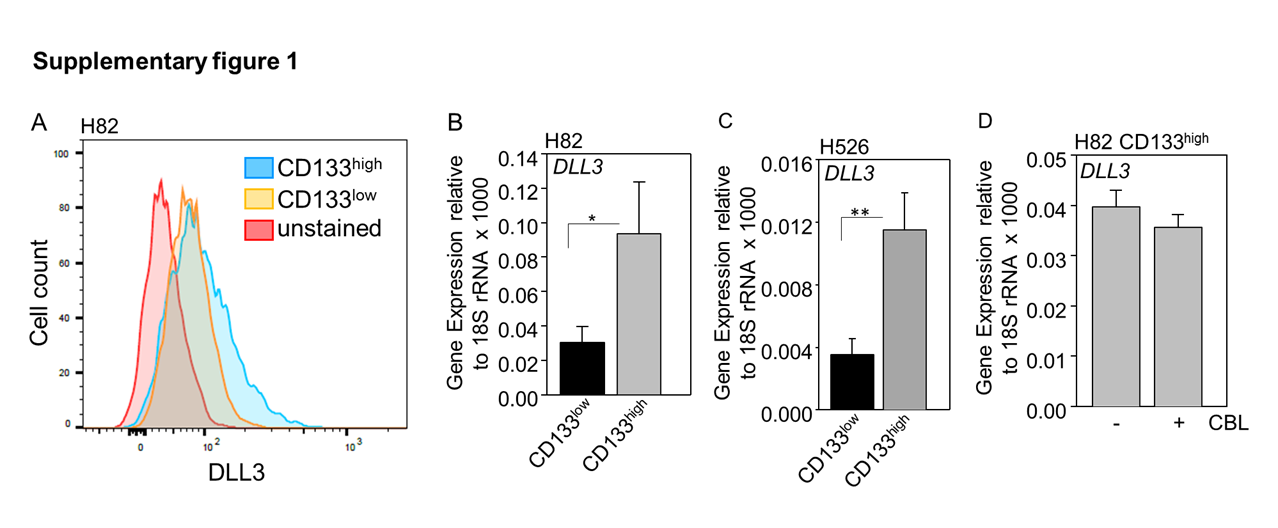


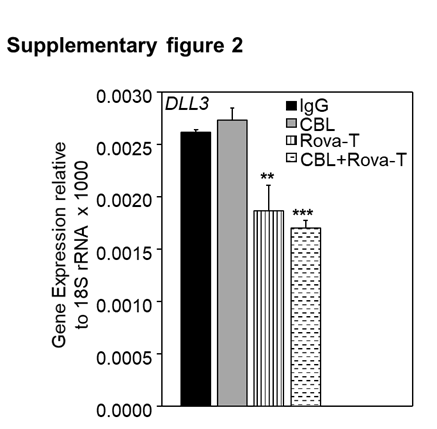


**Supplementary figure legend**

**Supplementary Fig. 1. High DLL3 expression in SCLC TICs is not decreased by CBL.** Expression levels of DLL3 on the cell surface of TICs and non-TICs derived from H82 cells were determined by FACS (**A**) and the levels of *DLL3* mRNA were determined by qPCR in the TICs and non-TICs of H82 (**B**) and H526 (**C**) cells. Expression levels of *DLL3* mRNA in TICs after treatment with CBL (1 µM) for 24h **(D)**. * *P< 0.05.*

**Supplementary Fig. 2. Rova-T treatment decreases DLL3 expression in SCLC tumours.** Expression levels of *DLL3* mRNA in residual tumours after treatment with vehicle for CBL+ IgG control, or with CBL, Rova-T, or Rova-T+CBL. **, *P< 0.01, ***, P< 0.001,* compared to control.
